# Non-linear pressure/temperature-dependence of high pressure thermal inactivation of proteolytic *Clostridium botulinum* type B in foods

**DOI:** 10.1371/journal.pone.0187023

**Published:** 2017-10-26

**Authors:** Maximilian B. Maier, Christian A. Lenz, Rudi F. Vogel

**Affiliations:** Lehrstuhl für Technische Mikrobiologie, Technische Universität München, Freising, Germany; University of Connecticut, UNITED STATES

## Abstract

The effect of high pressure thermal (HPT) processing on the inactivation of spores of proteolytic type B *Clostridium botulinum* TMW 2.357 in four differently composed low-acid foods (green peas with ham, steamed sole, vegetable soup, braised veal) was studied in an industrially feasible pressure range and temperatures between 100 and 120°C. Inactivation curves exhibited rapid inactivation during compression and decompression followed by strong tailing effects. The highest inactivation (approx. 6-log cycle reduction) was obtained in braised veal at 600 MPa and 110°C after 300 s pressure-holding time. In general, inactivation curves exhibited similar negative exponential shapes, but maximum achievable inactivation levels were lower in foods with higher fat contents. At high treatment temperatures, spore inactivation was more effective at lower pressure levels (300 vs. 600 MPa), which indicates a non-linear pressure/temperature-dependence of the HPT spore inactivation efficiency. A comparison of spore inactivation levels achievable using HPT treatments versus a conventional heat sterilization treatment (121.1°C, 3 min) illustrates the potential of combining high pressures and temperatures to replace conventional retorting with the possibility to reduce the process temperature or shorten the processing time. Finally, experiments using varying spore inoculation levels suggested the presence of a resistant fraction comprising approximately 0.01% of a spore population as reason for the pronounced tailing effects in survivor curves. The loss of the high resistance properties upon cultivation indicates that those differences develop during sporulation and are not linked to permanent modifications at the genetic level.

## Introduction

Low-acid (LA), shelf-stable foods are of special interest regarding the prevention of foodborne botulism caused by proteolytic *Clostridium* (*C*.) *botulinum* strains belonging to the physiologic group I of this heterogeneous species [1]. This is related to the absence of common hurdles preventing growth and the production of botulinal neurotoxins (BoNT) in such food products, i.e., storage temperatures < 10°C, pH values < 4.6 and water activity values < 0.93 [2]. Consequently, the primary causes for foodborne BoNT intoxications are insufficient sterilization and the ineffectiveness of other growth-inhibiting measures, which facilitate the survival of *C*. *botulinum* spores, their germination, outgrowth and subsequent toxin production [2].

The current practice of thermal processing to inactivate (*C*. *botulinum*) spores and achieve commercial sterility of LA foods is still based on the 12-D concept, i.e., the application of a thermal process sufficient to theoretically result in a 12-log cycle reduction in the numbers of viable spores. However, the application of harsh thermal treatments provokes a significant decrease in quality and often results in overprocessing of food products [3]. A promising alternative technique to inactivate spores and ensure the safety of LA foods is the combination of high hydrostatic pressure levels with elevated temperatures. The rationale behind this merge of technologies is to use potential synergistic effects to reduce spore counts more efficiently. Thereby, initial spore counts should be decreased to comparable levels as are achieved by traditional retort heating. Additionally, the total thermal load applied on the product can be reduced [4].

Accelerated but varying inactivation results for spores from different strains of *C*. *botulinum* by high pressure thermal (HPT) processing have been reported by several authors [5–10]. Consequently, no coherent inactivation strategy could be derived for spores of *C*. *botulinum* so far due to their diverse behavior towards HPT processing. This inconsistency becomes most apparent when considering the findings of Margosch et al. [5] for proteolytic *C*. *botulinum* strain TMW 2.357. There it has been demonstrated that HPT treatments at 600 and 800 MPa in combination with temperatures of 100, 110 and 120°C resulted in a reduced spore inactivation compared with equivalent thermal treatments at ambient pressure. These findings indicated a pressure stabilization of a spore fraction within the population during isobaric/isothermal holding times. Modeled isoeffect lines suggest that this effect of spore stabilization also occurs at pressure levels below 600 MPa. However, experimental data in a lower pressure range, which could verify the accuracy of this model or disprove it, are missing. Additionally, it is difficult to predict from existing data, whether stabilization effects also occur in relevant LA food matrices. Due to the importance of *C*. *botulinum* for food safety the lack of such data presents a fundamental gap of knowledge that has to be closed to come towards a precise evaluation of the safety of HPT processes for the production of shelf-stable LA food products.

This study contributes to closing this knowledge gap providing data for the HPT inactivation of proteolytic *C*. *botulinum* spores in four different LA ready-to-eat (RTE) foods. This HPT inactivation data are contrasted with the effectiveness of a standard retorting process. Furthermore, two questions were targeted that help to gain a better understanding of the nature of spore stabilization effects: (i) whether tailing effects depend of initial spore counts, i.e., whether they occur due to (detection) limits in the experimental design or due to the presence of resistant spore fractions, and (ii) whether surviving spores retain their resistance properties upon cultivation.

## Materials and methods

### Microorganism, growth conditions and spore production

The proteolytic *C*. *botulinum* type B strain TMW 2.357 (REB 89, obtained from the Institut für Medizinische Mikrobiologie und Infektionsepidemologie, Leipzig, Germany) was used in this study. This strain has been chosen for HPT studies because it can be considered as “worst case” as its spores exhibit extensive pressure resistance compared to other strains of *C*. *botulinum*, which are available to us [10]. Growth conditions and spore purification were basically performed as previously described by Lenz et al. [11]. Briefly, spore production started by inoculating 45 mL of tryptone-peptone-yeast extract-carbohydrates (4 g/L glucose, 1 g/L maltose, 1 g/L starch, 1 g/L cellobiose) (TPYC) broth [12] [11] with a -80°C glycerol stock culture and subsequent incubation in an anaerobic chamber (85% N2, 10% CO2, 5% H2) for 24 h at 37°C. The growing culture was then transferred into 450 mL of fresh TPYC broth and anaerobically incubated for 12 ± 2 d at 37°C. The produced spores were harvested by centrifugation (10.000 x g, 4°C, 10 min), washed three times with ice-cold deionized water and one time with S+ (0.85% saline + 0.1% Antifoam B Emulsion (Dow Corning, Germany) to reduce possible spore agglomeration) followed by incubation in 50% ethanol for 2 h at room temperature. Afterwards the spore suspension was washed for at least three more times with ice-cold deionized water and, finally, resuspended in deionized water obtaining a viable spore count of 108–10^9^ spores/mL. Sporulation resulted in a uniform population of phase bright spores ≥ 90% as determined by phase contrast microscopy. The spore suspensions were stored at 4°C until use.

### Sample preparation

The heat-sterilized RTE foods used for inactivation studies were vegetable soup (VS), green peas with ham (GPH), steamed sole (SS) and braised veal (BV). A detailed composition of the four LA food products is given in Table 1. Before use, the food samples were blended to give homogeneous pastes. Afterwards, the food samples were inoculated with the spore suspension (ratio 1:100) and thoroughly mixed with a spatula and vortexing. For HPT experiments, the inoculated food samples were filled into custom-made PTFE tubes and closed with silicon stoppers that were fastened by screw-caps. For thermal treatments, samples were prepared in the same manner, but samples were filled into custom-made stainless steel tubes. This allowed more rapid heating and cooling rates. In addition, uninoculated samples were prepared for temperature profile measurements in the geometrical center of the vial during processing. All samples were stored on ice before and after treatments.

**Table 1:** Food product formulations.

Food product	Fat [wt%]	Protein [wt%]	Carbohydrates [wt%]	Salt [wt%]	Fiber [wt%]	pH	a_w_
**Green peas with ham**	12.8	5.1	5.1	1.1	3.9	6.0	0.973
**Steamed sole**	8.6	10.2	5.7	2.9	1.7	6.75	0.979
**Braised veal**	6.2	7.6	6.7	3.7	1.5	6.53	0.973
**Vegetable soup**	2.9	1.2	5.9	1.0	1.3	5.8–6.0	0.98

wt%, percentage by weight

### High pressure equipment and treatment

**Fig 1:**
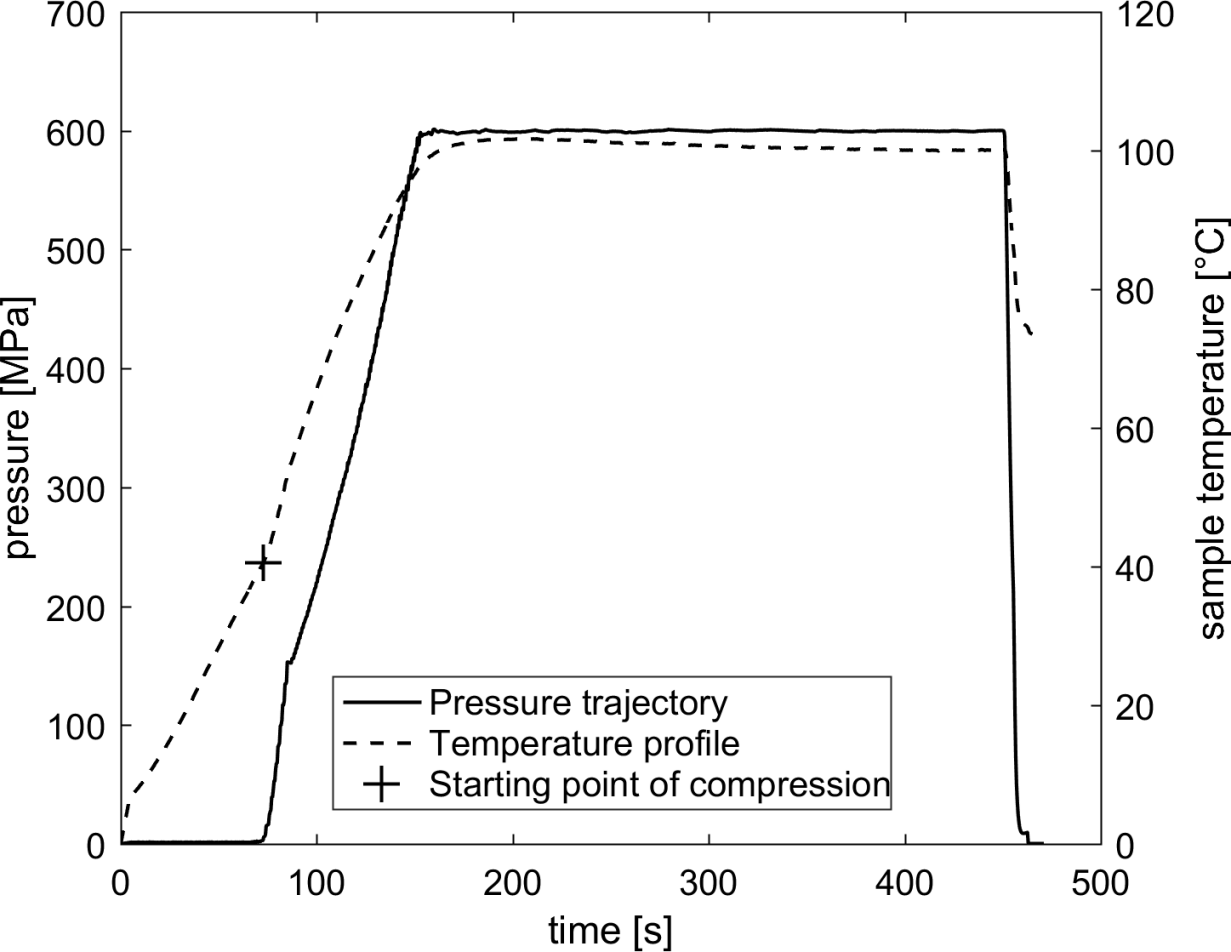
Typical temperature and pressure profiles during HPT treatments. Exemplarily, pressure (dashed line) and temperature (solid line) profiles of a HPT treatment with a target pressure of 600 MPa, a target temperature of 100°C and a pressure-holding time of 300 s are depicted. A cross marks the starting point of compression (40.5°C, 72.5 s).

The HPT equipment consisted of a hand pump-driven high pressure intensifier system (Unipress, Warsaw, Poland). With an inner volume of 8 mL, two samples could be placed inside the high pressure vessel (type MV2-13, Unipress, Warsaw, Poland) at a time. Bis(2-ethylhexyl) sebacate (Nr. 84822; Sigma-Aldrich, USA) served as a pressure transmitting fluid. The double wall high pressure vessel was temperature-controlled by a circulating oil bath (witeg Labortechnik GmbH, Wertheim, Germany) with silicon oil (Sil 180, Fisher Scientific, New Hampshire, USA) as a heating fluid. The lid of the high pressure vessel was equipped with a lead-through for a type K thermocouple. To achieve isobaric/isothermal pressure-holding times, the start of compression began at an empirically determined target pressure, target temperature, and sample type-dependent temperature. This starting point is shown exemplarily in Fig 1. The temperature was monitored in the geometrical center of an uninoculated sample vial directly above the inoculated sample. Pressure levels applied ranged from 300 up to 600 MPa, process temperatures from 100 to 120°C and pressure-holding times from 1 to 300 s. The average compression rate was around 6.5 MPa/s and decompression took place in less than 12 s.

### Thermal treatments

Thermal inactivation experiments (121.1°C, 3 min) were performed similar to the HPT experiments with the exception that samples were not pressurized and stainless steel sample tubes were used to allowed rapid heating and cooling.

### Enumeration of surviving spores

Surviving spores were enumerated by pour-plating with TPYC agar (15 g/L agar–agar) immediately after treatment. Samples were opened in an anaerobic chamber (85% N_2_, 10% CO_2_, 5% H_2_), serially diluted in S+ solution and pour-plated in duplicate. Survivors were counted after anaerobic incubation for up to 5 d at 37°C. For visualization, the results are presented as log_10_ (N/N_0_), where N describes the number of surviving spores after a treatment and N_0_ is the initial spore count.

### Statistical analysis

All experiments were conducted at least in independent duplicates. The significance of differences between mean values from impendent experiments was determined by one-way ANOVA. Tukey’s HSD test at an error probability of 5% (P < 0.05) served as a post-hoc analysis. Statistical analysis was performed using MATLAB software (version R2016B, Mathworks, Natick, USA).

## Results

### High pressure thermal inactivation in food

**Fig 2:**
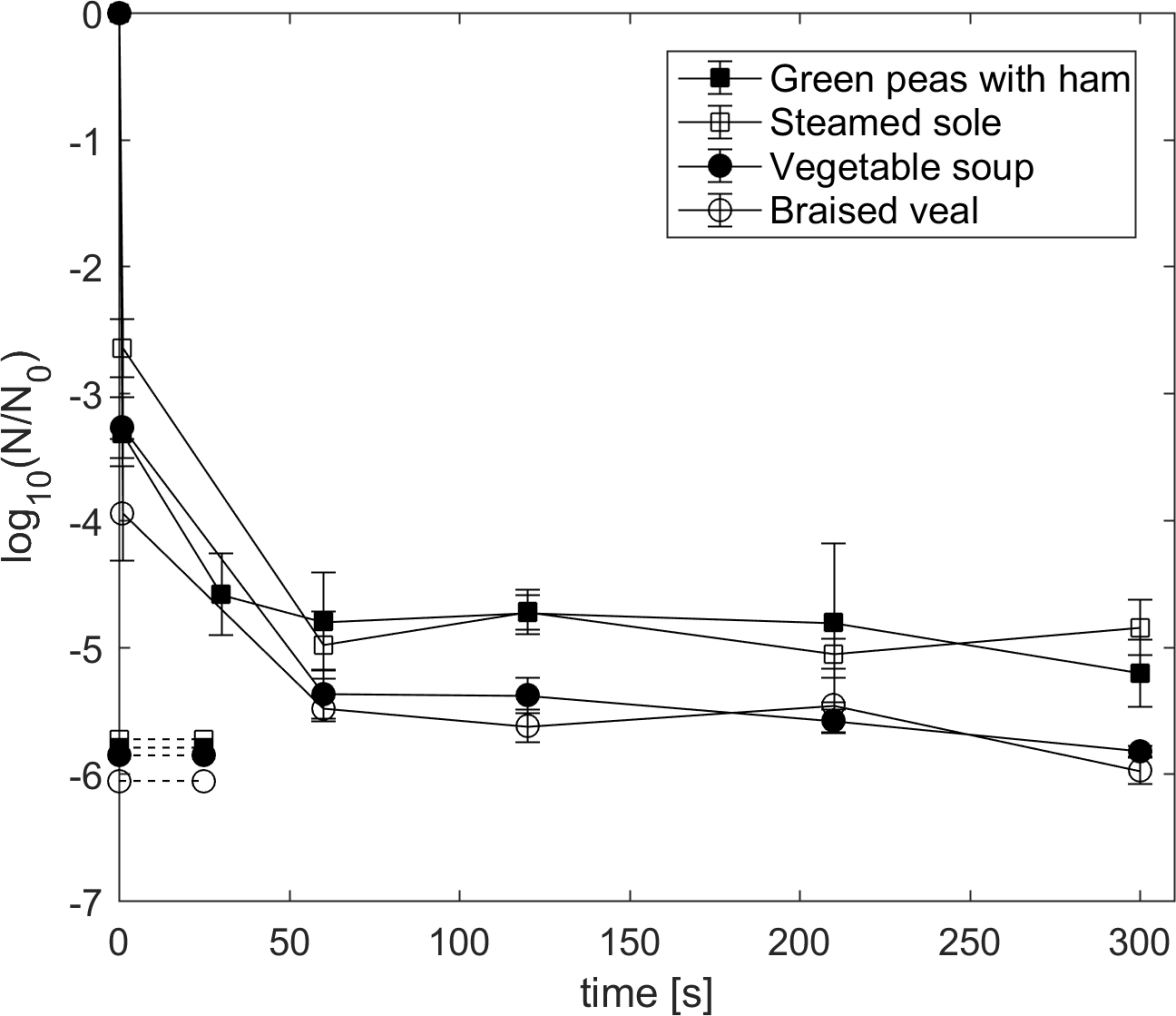
Log reduction of *C*. *botulinum* TMW 2.357. Spores suspended in green peas with ham (solid squares), steamed sole (open squares), vegetable soup (solid circles) and braised veal (open circles) after HPT treatment at 600 MPa and 110°C for 1 to 300 s. Dashed lines indicate the corresponding detection limits. Initial spore count: 10^7^ spores/g. Data are shown as means ± standard deviations of triplicate independent experiments.

The HPT treatments at 600 MPa and 110°C resulted in rapid spore inactivation in all four RTE foods (green peas with ham, GPH; steamed sole, SS; vegetable soup, VS; braised veal, BS) within the first 60 s (Fig 2). A minimum log reduction of 2.6 log units (SS) and a maximum of 3.9 log units (BV) was achieved after a pressure-holding time of 1 s, i.e., after compression and instant decompression. Isobaric/isothermal pressure-holding times between 60 s and 300 s did not result in further inactivation in all four food matrices. Spores tended to be more resistant in SS and GPH, reaching final inactivation levels of 4.8 and 5.2 log units, respectively. On the contrary, spores treated in VS and BV were significantly less resistant (p < 0.05) to pressure treatments for 300 s, with log reduction values of 5.8 and 6.0 in VS and BV, respectively. At this final time point, minimum one out of three viable spore counts reached the detection limit (VS: -6.0; BV: -5.9-log cycle reduction in CFU). Since, along with SS, spores of *C*. *botulinum* TMW 2.357 showed greatest resistance in GPH, and outbreaks of foodborne botulism connected to proteolytic *C*. *botulinum* type B are often linked to food products involving ham and vegetables [2], further inactivation experiments were conducted in GPH.

### High pressure thermal inactivation in GPH

**Fig 3:**
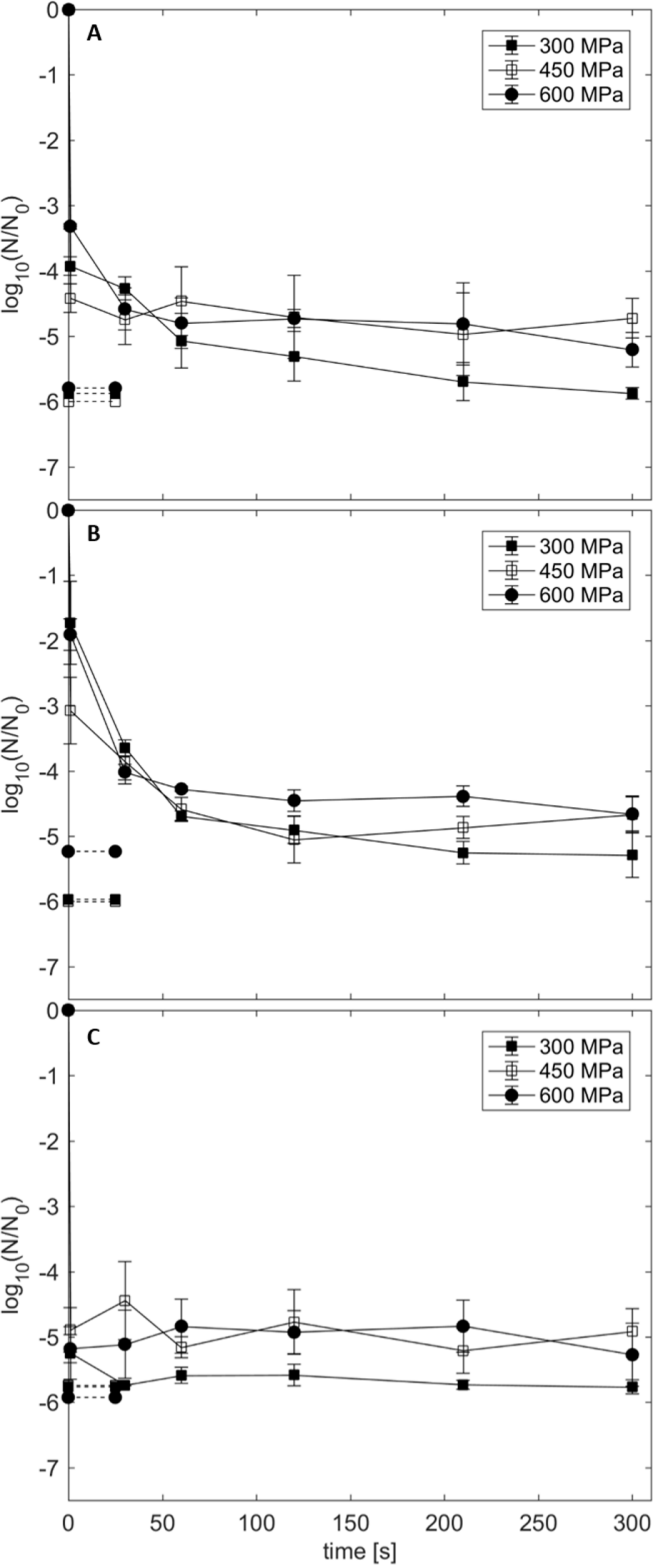
Log reduction of C. botulinum TMW 2.357 spores in green peas with ham. Target pressure levels of 300 MPa (solid square), 450 MPa (open square) and 600 MPa (solid circle) in combination with process temperatures of 100°C (A), 110°C (B) and 120°C (C) were applied for 1–300 s. Dashed lines indicate the corresponding detection limits. Initial spore count: 10^7^–10^8^ spores/g. Data are shown as means ± standard deviations of triplicate independent experiments.

To evaluate the impact of HPT processing in an industrial and economical feasible pressure range, pressure levels of 300, 450 and 600 MPa in combination with 100, 110 and 120°C were applied on spores in GPH for up to 300 s (Fig 3). A considerable effect on spore inactivation was observed after compression and decompression (1 s pressure-holding time) at all pressure/temperature combinations. This effect increased with increasing process temperatures, having its maximum impact at 120°C. Prolonged pressure-holding times (> 1 s) exhibited reduced spore inactivation, where differences became less pronounced with increasing process temperatures. Hence, isobaric/isothermal pressure-holding times had only marginal impact on further spore inactivation, especially for process temperatures ≥ 110°C. Remarkably, a combination of 300 MPa and 100, 110 or 120°C resulted in higher inactivation levels than pressure levels of 450 and 600 MPa and the same process temperatures. At a pressure level of 300 MPa in combination with 120°C and 110°C, the detection limit could already be reached after 30 s and after 210 s, respectively. Treatments at pressure levels > 300 MPa were not sufficient to reduce the spore count below the detection limit at any pressure/temperature combination. For example, the maximum inactivation after 300 s pressure-holding time at 120°C was 4.9 log units at 450 MPa and 5.3 log units at 600 MPa. Thus, spores of *C*. *botulinum* TMW 2.357 exhibited increased HPT resistance at pressure levels > 300 MPa resulting in pronounced tailing effects.

### Thermal inactivation at ambient pressure

Thermal treatments of ~10^8^ spores/g *C*. *botulinum* TMW 2.357 spores in GPH at 121.1°C for 3 min resulted in an average of 5.4 ± 0.2 log cycles reduction. The detection limit of 6.7-log cycle reduction was not reached in any of the three experiments conducted. This indicates that the theoretical 12-log cycle reduction is not achievable by this standard heat sterilization treatment assuming a D-value of 0.25 min (green pea soup; [13]).

### Impact of initial spore count on HPT inactivation

**Fig 4:**
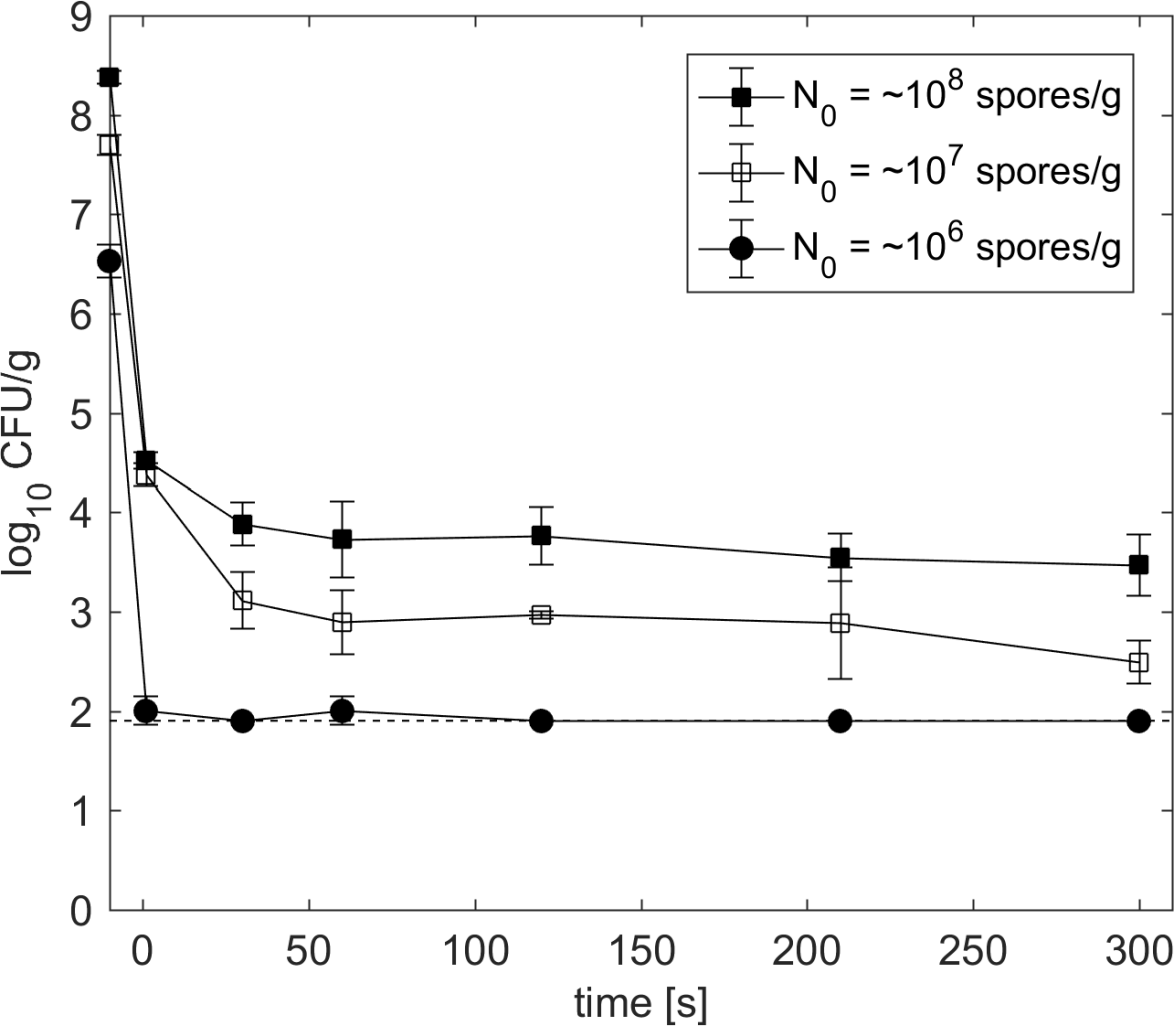
Viable spores (CFU/g) of *C*. *botulinum* TMW 2.357 at 600 MPa and 110°C in green peas with ham. Different symbols indicate different initial spore counts, i.e., 10^6^ (circles), 10^7^ (open squares) and 10^8^ (solid squares) spores/g. The dashed line represents the detection limit (log_10_ CFU/g: 1.9). Data are shown as means ± standard deviations of triplicate independent experiments.

The impact of varying initial spore counts on the inactivation at 600 MPa and 110°C is shown in Fig 4. The curve shapes of the inactivation kinetics were similar and only shifted along the y-axis depending on the initial spore count. A minimum reduction to 4.5 log CFU/g was achieved after 30 s of pressure-holding time independent of the initial spore count. Longer isothermal/isobaric holding times resulted in slow inactivation. With initial spore counts of 10^6^ CFU/g, the detection limit was reached within 1 s. However, inactivation curves starting with 10^7^ and 10^8^ CFU/g indicate that approximately one out of 10,000 spores survives HPT treatments up to 300 s.

### High pressure thermal treatment of surviving fraction

**Fig 5:**
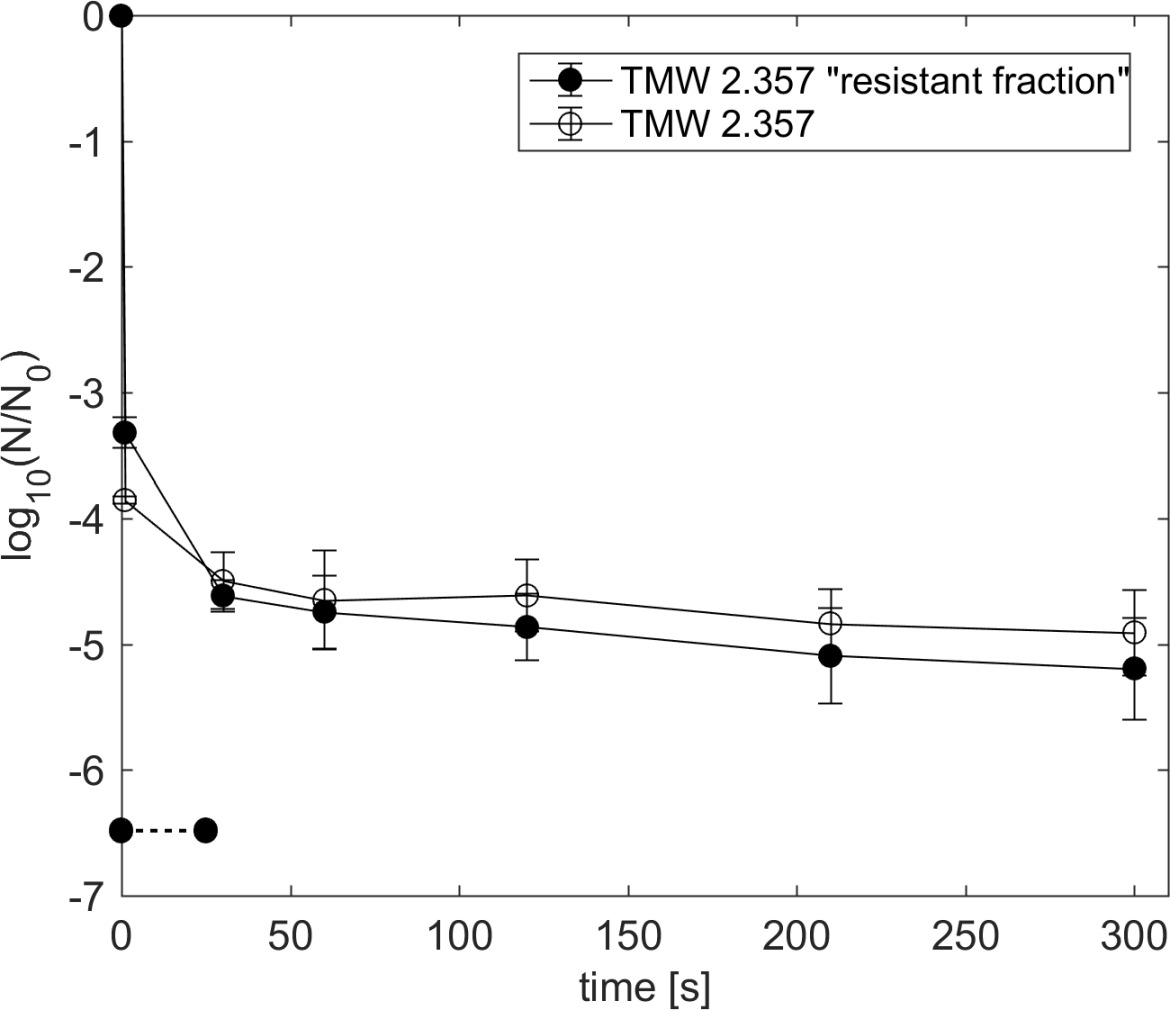
Inactivation of *C*. *botulinum* TMW 2.357 spores at 600 MPa and 110°C in green peas with ham. Solid circles indicate spores produced from the resistant fraction surviving HPT treatment at 600 MPa, 110°C for 300 s. Open circles indicate spores produced using the standard procedure. Data are shown as means ± standard deviations of at least duplicate independent experiments.

Spores that survived HPT treatments at 600 MPa, 110°C for 300 s were picked from agar plates and used to produce a fresh spore suspension. The comparison of the HPT resistance of spores grown from glycerol stocks (standard procedure) and spores produced from the HPT resistant fraction is shown in Fig 5. No significant differences in the HPT resistance of both spore batches was observable. Rapid inactivation took place within the first 60 s and final (300 s) log reduction values were around 5 log units for both spore batches. Tailing of the inactivation curves occurred in the same way as observed before.

## Discussion

To date, few studies investigated the HPT inactivation of spores from the important food intoxicating organism, *C*. *botulinum*, in food matrices and food model systems [7–10, 14]. Moreover, the comparison of inactivation data is not only difficult due to varying process parameters but also because of different thermodynamic properties of HPT units, in particular controlling and monitoring adiabatic heating effects. Data reported here extend the knowledge of HPT inactivation of a relatively resistant proteolytic *C*. *botulinum* type B strain (TMW 2.357; [5, 10]) in relevant matrices, i.e., low-acid ready-to-eat (LA RTE) foods under matrix-independent isothermal isobaric conditions.

In principle, HPT treatments at 600 MPa and 110°C resulted in similar curve shapes in all four LA foods whereby spores treated in GPH (green peas with ham) and SS (steamed sole) showed greater resistance. Bull et al. [8] found that spores of proteolytic *C*. *botulinum* exhibited increased heat tolerance when treated in a model product with a higher fat content (~7.4% fat). However, this effect was less distinct when spores were HPT-treated. For spores of non-proteolytic *C*. *botulinum* type E, it has been shown that increasing fat content can result in reduced HPT and thermal inactivation [14, 15]. Both GPH and SS have a higher fat content than BV (braised veal) and VS (vegetable soup), namely 12.8% and 8.6%, respectively. From the current setup it is impossible to spot specific food components that are responsible for the slightly different inactivation results in the different RTE foods. However, results obtained here together with previously published data suggest that, besides other food components, the overall fat content and/or probably the fat composition are likely to play some role in the HPT inactivation of *C*. *botulinum* spores.

Previous studies showed the increased HPT resistance of proteolytic *C*. *botulinum* TMW 2.357 spores in a pressure range between 600 and 1400 MPa [5, 10]. Since the maximum pressure that is applied in commercial operations is about 600 MPa [16], the focus of this study was on equal or lower pressure levels. Our results complement and, in general, support the findings reported by Margosch et al. [5] even though earlier experiments were conducted in a different HPT unit, spores were treated in Tris-His buffer and sporulation conditions were different. We could also observe drastically decreased inactivation rates at longer isothermal/isobaric holding times, i.e., a significant tailing effect in survivor curves. Such tailing effects appear to be significantly less pronounced for *C*. *botulinum* strains belonging to the non-proteolytic group II [14, 17]. Most importantly, this demonstrates that it can be difficult to completely inactivate spores from proteolytic *C*. *botulinum* strains in relevant food matrices by HPT treatments in an industrially feasible range, and that an extension of the holding time is not always a suitable approach to reach commercial product sterility. Interestingly, the efficiency of spore inactivation increased applying lower pressure levels of 300 MPa at process temperatures of 100, 110 and 120°C. This, in turn, demonstrates the non-linearity of the effectiveness of different pressure levels and the importance of finding effective p/T combinations when designing HPT processes to inactivate proteolytic *C*. *botulinum* strains. At 300 MPa, inactivation results with equal or even higher log values (≥ 5.4 log units) than after standard thermal sterilization treatments at ambient pressure (121.1°C, 180 s) became achievable. This was the case at 110°C within 210 s and at 120°C within 30 s. This demonstrates the potential of replacing standard retorting processes by HPT processes achieving product safety at lower process temperatures and/or shorter treatment durations.

Based on the widely accepted parameters for heat sterilization (D_121.1 °C_ ≈ 0.21 min, z = 10°C) [18–21], the thermal treatment applied (121.1°C, 3 min) should have resulted in a theoretical inactivation of > 14-log cycles of *C*. *botulinum* spores (12 log when 0.25 min are used for calculation), which is far away from the achieved 5.4-log cycles for *C*. *botulinum* TMW 2.357 in GPH. This is not surprising since thermal log inactivation curves can be non-linear and, in addition to matrix effects, large strain-dependent differences in the heat resistance of *C*. *botulinum* spores were reported, i.e., D_120 °C / 121.1 °C_ values of up to 1.2 min [10, 22]. In line with previous argumentations [5, 10], this indicates that the 12-D concept generally recommended for the inactivation of *C*. *botulinum* in shelf-stable LA RTE food is in fact only a 4-D concept for resistant *C*. *botulinum* spores. The fact that standard heat sterilization processes are sufficient to ensure proper consumer safety is putatively mainly related to relatively low contamination levels of *C*. *botulinum* in food samples in general (10 to 1000 spores/kg) [23, 24].

Since all obtained HPT inactivation curves exhibited tailing effects subsequent to a generally high inactivation effect within the first seconds, experiments with varying initial spore counts were conducted. One major reason was to exclude that inaccuracies as cell counts approach the detection limit play a role in the observed tailing effects. Generally, survivor curve tailing has been frequently reported, but the exact reasons still remain unclear. Proposed contributing factors include heterogeneous resistance properties within a spore population, spore clumping, adhesion to any surfaces during sample handling and protective effects of dead spores [3, 5, 25–27]. Microscopic analysis of aqueous spore suspensions indicated that there is no great tendency of *C*. *botulinum* spores to aggregate, which could be observed regardless of the spore concentration. Although it cannot be excluded that this is different in the food matrices used, results suggest that tailing effects are related to the presence of a small resistant spore fraction accounting for approximately 0.01% of a spore population.

The fact that spores produced from HPT survivors exhibited similar inactivation kinetics as spores produced from glycerol stocks indicates that the resistance properties of the resistant spore fraction are not related to intrinsically different properties, e.g., due to genetic variations.

Our results suggest that HPT processes based on the maxime “the more pressure, the better” are not always the best choice and should be kept in mind for future HPT process design and food safety considerations. While non-linear pressure/temperature-dependence of the HPT spore inactivation efficiency is likely also for other strains, respective quantitative effects may differ. Therefore, more HPT inactivation studies with other, differently pressure (and heat) resistant strains of *C*. *botulinum* are necessary to draw any general conclusion on how the phenomenon observed for the strain used in this study affects the overall suitability of HPT for commercial implementations. Regarding this, a closer look on the impact of single food components, e.g. fatty acids or proteins could also contribute to a more generic approach towards the inactivation of spores of *C*. *botulinum*.
